# Advances in Functional Biopolymer-Based Nanocomposites for Active Food Packaging Applications

**DOI:** 10.3390/polym13234198

**Published:** 2021-11-30

**Authors:** Nagaraj Basavegowda, Kwang-Hyun Baek

**Affiliations:** Department of Biotechnology, Yeungnam University, Gyeongsan 38541, Gyeongbuk, Korea; nagarajb2005@yahoo.co.in

**Keywords:** biopolymer, nanocomposites, shelf life, food packaging, antimicrobial

## Abstract

Polymeric nanocomposites have received significant attention in both scientific and industrial research in recent years. The demand for new methods of food preservation to ensure high-quality, healthy foods with an extended shelf life has increased. Packaging, a crucial feature of the food industry, plays a vital role in satisfying this demand. Polymeric nanocomposites exhibit remarkably improved packaging properties, including barrier properties, oxygen impermeability, solvent resistance, moisture permeability, thermal stability, and antimicrobial characteristics. Bio-based polymers have drawn considerable interest to mitigate the influence and application of petroleum-derived polymeric materials and related environmental concerns. The integration of nanotechnology in food packaging systems has shown promise for enhancing the quality and shelf life of food. This article provides a general overview of bio-based polymeric nanocomposites comprising polymer matrices and inorganic nanoparticles, and describes their classification, fabrication, properties, and applications for active food packaging systems with future perspectives.

## 1. Introduction

Nanoscience and nanotechnology have matured into extremely active, vital, and expanding fields of research for developing small particles with multidimensional applications in the areas of nutrition, agriculture, cosmetics, paints and coatings, personal care products, catalysts, energy production, lubricants, security printing, molecular computing, structural materials, drug delivery, medical therapeutics, pharmaceuticals, and diagnostics [1]. The remarkably small size of these materials provides a large surface-area-to-volume ratio and, consequently, more surface atoms compared to their microscale counterparts. This improves the properties of materials with negligible defects on their surfaces [2]. Moreover, nanomaterials have been developed as nanocomposites, which are engineered solid materials that result when two or more different constituent materials with different physical and chemical properties are combined to create new substances [3]. Nanocomposites are hybrid materials consisting of mixtures of polymers and inorganic solids (such as clays and oxides) at the nanometer scale. The remarkably complicated structure of nanocomposites, in which one phase (such as nanoparticles (NPs) and nanotubes) has a nanoscale morphology, exhibits properties that are superior to those of microcomposites in an assembled structure [4].

Polymeric nanocomposites are produced by dispersing NPs or nanofillers into polymeric matrices. This reinforcement results in a matrix with unique, enhanced physical and mechanical properties [5]. The combination of these two materials produces a synergistic effect with special properties that are not exhibited by the individual components. Moreover, the preparation of nanocomposites with more than two components effectively assists in satisfying the design and strength requirements for specific applications [6]. Inorganic NPs have unique properties such as mechanical, magnetic, electrical, and catalytic characteristics. On the other hand, polymers are assemblies of different monomers with properties such as low weight, flexibility, and low-cost production. Combining these two substances yields novel, unique materials with high performance and unusual, incomparable properties [2]. Polymeric nanocomposites have attracted considerable attention because of their unique and enhanced mechanical, optical, thermal, diffusion barrier, magnetic, and electric properties compared with those of micro-, conventional, and individual components [7]. These unique and incomparable properties of polymeric nanocomposites and their synergistic multifunctions, achieved through the incorporation of multiple components into one compatible entity, have led to their broad application in different fields [8].

Biopolymers or biodegradable polymers are renewable natural resources generated from biological systems, such as plants, animals, and microorganisms, and/or chemically synthesized from the starting materials of natural fats or oils, sugars, and starch [9]. Natural biopolymers are alternatives to synthetic polymers obtained from non-renewable petroleum resources. Therefore, biodegradable polymers can be disintegrated or degraded by the enzymatic action of specific microorganisms, organic byproducts, methane, inorganic compounds, biomass, carbon dioxide, and water [10]. Biopolymers offer several advantages, such as low-cost extraction, biocompatibility, biodegradability, environmental friendliness, and lack of environmental toxicity. Therefore, biopolymers have been traditionally used in various industrial activities related to the biomedical [11], pharmaceutical [12], food [13], and environmental sectors [14]. Examples of biopolymers include protein isolates (soy, wheat, corn, gluten, whey, and gelatin), carbohydrates (pullulan and curdlan), polysaccharides (chitosan, alginates, starch, and cellulose derivatives), and lipids (bees, wax, and free fatty acids) [15]. Additionally, polylactic acid (PLA), polyvinyl alcohol (PVA), polycaprolactone (PCL), polyhydroxybutyrate (PHB), polybutylene succinate (PBS), and their blends are some examples of synthetic biopolymers [16].

Food packaging is a particularly critical step in the protection and preservation of food to ensure its safety and increase its shelf life. This factor plays a key role in the global food industry in terms of satisfying consumer demand for safe, fresh, high-quality, durable, and healthy food, along with addressing certain challenges such as cost efficiency, environmental issues, consumer convenience, and food safety regulations [17]. Food packaging has a crucial function in the modernized food industries, as package performs a sequence of tasks—primarily containment, safety, handiness, and communicating information. These functions must be evaluated and considered simultaneously during the packaging and development process, as they are all interconnected [18]. Therefore, the food industry is constantly in pursuit of novel and alternative technologies for improving critical parameters such as the quality, safety, and shelf life of food products. Food packaging is primarily used to protect food products against adverse external environmental elements, including microorganisms, heat, light, oxygen, moisture, enzymes, pressure, insects, dust, and dirt [19]. In addition, packaging provides tampering resistance, physical support, and chemical or biological requirements. It also prevents spoilage and contamination, and increases sensitivity by enabling the enzyme activity of food products in the process of storage, transport, and distribution [20]. The incorporation of functional nanomaterials into biopolymer matrices improves the physicochemical properties [21], mechanical and barrier properties, moisture stability, durability, and flexibility of food packaging materials. Moreover, this blending improves active and smart/intelligent packaging functions, such as antimicrobial, antioxidant, UV protective, and nanosensing characteristics for the detection of small organic molecules and gases [22].

Biopolymer-based nanocomposites are a new type of material that exhibit considerably enhanced properties such as barrier, mechanical, and thermal characteristics, and have been considered as novel and alternative packaging materials [23]. Biopolymer nanocomposites are bio-based multiphase materials composed of two or more constituents, in which the continuous phase (matrix) is a biopolymer, and the discontinuous phase (fillers) is composed of NPs. These packaging materials can interact with food by releasing certain active substances, such as antimicrobial and antioxidant agents, or by removing unfavorable elements such as water vapor and oxygen [24]. This review presents a systematic assessment of the most recent advances in research on the development of polymeric nanocomposites for food packaging systems based on their classification, fabrication methods, properties, and applications. In addition, the review highlights antimicrobial activities, the related mechanisms of action, and future perspectives for ensuring the safety of nanomaterials for active food-packaging systems. The development of smart (active/intelligent) food packaging materials using bio-based nanocomposite materials can play an important role not only in minimizing environmental issues but also in enhancing the functions of food packaging systems.

## 2. Polymer Nanocomposites

Composite materials are produced by combining two or more distinct constituents or phases with different physical or chemical properties [25]. Although the different materials do not blend into each other, they yield unique properties (mechanical, physical, thermal, and electrical) in a supplementary manner upon being composited and engineered into a complex architecture at the micro- or macro-scale levels [26]. Composite materials typically comprise two phases: a continuous phase (matrix) and a discontinuous phase (reinforcements). Matrix materials (ceramics, metals, or polymers) are responsible for maintaining the positions of the reinforcement materials, whereas the filler materials (fibers and particles) impart new properties to the matrix phase [26]. Polymers have gained significant attention owing to their unique properties such as low cost, high flexibility, low weight, high strength, specific stiffness, biocompatibility, and ease of production [27]. Nanocomposites or nanofillers such as organic and inorganic materials, clay, and carbon nanostructures are used as coatings. Organic nanofillers include polymer nanofibers, natural fibers, and natural clay [27], and inorganic nanofillers include metals (such as Au, Ag, and Fe), metal oxides (CuO, ZnO, FeO, and TiO) [28], and carbon (fullerenes, graphene, carbon nanotubes, and nanofibers) [29].

Polymeric nanocomposites are a combination of a polymer matrix (continuous phase) and inorganic NPs (discontinuous phase) with at least one dimension at the nanometer scale. These composites exhibit improved properties compared to those of polymers, such as high strength, thermal stability, electrical conductivity, chemical resistance, flame retardancy, and optical characteristics [30]. Commonly used polymers in the food packaging industry, as either matrices or substrates for coatings, include synthetic low-density polyethylene (LDPE), high-density polyethylene (HDPE), polyethylene terephthalate (PET), polypropylene (PP), polyamide (PA), ethylene vinyl alcohol (EVOH), and polystyrene (PS). Naturally occurring biodegradable polymers or biopolymers, such as polyhydroxyalkanoates (PHAs), PHB, PLA, poly(hydroxybutyrate-*co*-hydroxyvalerate) (PHVB), polyvinyl alcohol (PVOH), and PCL, which are polyesters produced by numerous microorganisms and the bacterial fermentation of sugars or lipids, are also similarly employed [31]. Various types of nanofillers have been used to fabricate nanocomposites. The most commonly used nanofillers are clay nanomaterials (such as montmorillonite, kaolinite, halloysite, saponite, hectorite, and laponite), silica NPs, carbon nanotubes (CNTs), nanosheets, graphene, silver, copper, zinc, titanium dioxide, copper oxide, zinc oxide, cellulose nanofibers, starch nanocrystals, chitosan, and chitin whiskers [32,33,34]. Achieving a uniform and homogenous dispersion of NPs in polymer matrices is the key challenge in obtaining nanocomposites with desirable properties [35]. The uniform dispersion of nanofillers (nanoscale dispersion) can lead to a large interfacial area in the composite matrix. This reinforcement depends on several factors, such as particle size, distribution, orientation, structure, and the properties and concentrations of the polymer matrix and filler [36].

### 2.1. Classification of Polymer Nanocomposites

Polymer nanocomposites can be categorized based on the dimensions of nanofillers (0D, 1D, 2D, and 3D), type of nanofiller (metal and metal oxide, metal sulfide, metal hydroxide, and silicate), type of polymer matrix (thermoplastic, thermoset, elastomer, natural, and biodegradable polymer matrix), and synthesis methods (ex situ, in situ, and simultaneous polymerization).

#### 2.1.1. Classification Based on the Dimension of Nanofillers

**Figure 1:**
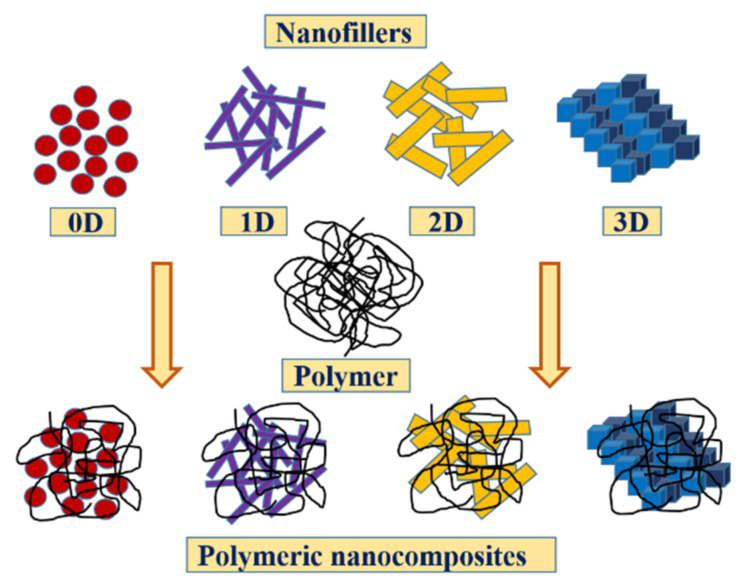
Classification of nanomaterials based on their dimensionality: zero- (0D), one- (1D), two- (2D), and three-dimensional (3D) nanocomposites.

Nanofillers can be categorized into zero- (0D), one- (1D), two- (2D), or three-dimensional (3D) nanoparticles according to their dimensions at the nanoscale (Figure 1).

Zero-dimensional (0D) nanofillers have dimensions that are all within the nano-range, such as NPs, that is, they do not have any dimension beyond 100 nm. These nanofillers may be amorphous, crystalline, metallic, ceramic, or polymeric in nature [37], and include materials such as gold, silver, and quantum dots (diameters in the range of 1–50 nm). One-dimensional (1D) nanofillers have a prominent dimension along one direction, and a nanostructure that is outside the nanometer range. These materials are long and have diameters of only a few nanometers [38]. These include nanotubes, nanorods, and nanowires of metals and metal oxides. Two-dimensional (2D) nanofillers have prominent dimensions in two directions and nanostructures that are outside the nanometer range. These large and extremely thin materials include nanofilms, nanosheets, nanowalls, nanofibers, and nanowhiskers; carbon nanotubes and montmorillonite are some of the noteworthy examples [39]. Three-dimensional (3D) nanofillers have dimensions along three directions, and nanostructures that are outside the nanometer range; however, the size of the individual blocks (structural units) is on the nanometer scale. These materials include nanoclays, nanogranules, and equiaxed NPs; zeolites are a notable example [40].

#### 2.1.2. Classification Based on the Types of Nanofillers

Metal or metal oxide NPs are homogenously dispersed or spread onto the polymer matrix to form a homogenous nanophase-separated structure that imparts the nanocomposites with flame retardancy and thermal stability. Silica, zinc, magnesium, titanium oxide, zirconium oxide, aluminum oxide, and iron oxide are typical examples in this regard [41]. Metal-sulfide/polymer nanocomposites have attracted considerable attention owing to their thermal, optical, electrical, and mechanical properties. Nanocomposites containing metal sulfide NPs incorporated into a polymer matrix exhibit enhanced thermal stability compared to that of pure polymers [42]; CdS, ZnS, and HgS are prominent examples of this type of nanofiller. Metal hydroxide NPs, such as aluminum and magnesium hydroxide NPs, are known to exhibit remarkable flame retardancy and thermal stability. Magnesia and alumina powders or double hydroxides, such as zinc and alumina, have been deposited as fillers on the surfaces of polymer matrices to prepare flame-retardant polymer nanocomposites [43]. Silicate/polymer nanocomposites exhibit decent mechanical and thermal properties. The direct mixing of silica into polymers is the simplest method for preparing these silicate/polymer nanocomposites. This mixing can be achieved by solution and melt blending to prepare flame-resistant silicate/polymer nanocomposites [44].

#### 2.1.3. Classification Based on Type of Polymer Matrix

As the name suggests, thermoplastic resins are polymers that can be molded/softened under pressure and heat, and hardened by cooling. Thermoplastic resins exhibit remarkable properties such, as high strength, high moldability, and chemical resistance, remolding, and recycling characteristics. For example, PS, PA, polyethylene (PE), and polyvinyl chloride (PVC) form thermoplastic networks [45]. Thermosetting polymers are generally liquid materials at room temperature and are the opposite of thermoplastics. They have three-dimensional covalent-bonded structures and, therefore, cannot be remolded by a heating–cooling process similar to that of thermoplastics. Moreover, these polymers harden irreversibly upon heating. Polyesters, vulcanized rubber, polyurethanes, and epoxy resins are examples of thermosetting polymers [46]. Elastomers, as their name suggests, exhibit elastic properties and can be stretched to a large extent without any damage. They are viscoelastic in nature because of their high viscosities and weak intermolecular forces. Polybutadiene, chloroprene, epichlorohydrin, and natural, silicone, and polyacrylic rubber materials are examples of elastomer matrices [47]. Biodegradable polymers or biopolymers are more soft and flexible than other polymeric materials. Starch, cellulose, chitosan, collagen, and proteins are the main sources of natural polymers, and PLA, PHB, and poly (3-hydroxybutyrate-co-3-hydroxyvalarate (PHBV) are typical examples of biopolymers.

### 2.2. Methods of Polymer Nanocomposite Preparation

Nanocomposites are prepared by incorporating nanofillers into polymer matrices to provide or improve the barrier, thermal, and mechanical properties of polymers. Polymer nanocomposites can be formulated by various approaches, including in situ polymerization, melt processing, and solution blending or casting methods.

#### 2.2.1. In Situ Polymerization

**Figure 2:**
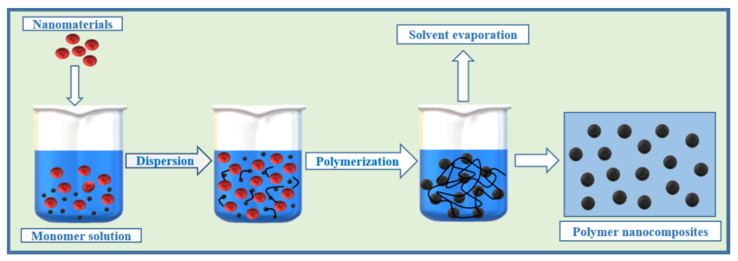
Schematic illustration of the in situ polymerization method.

This is an effective method that has been extensively used in the last few decades to prepare polymer nanocomposites. Typically, nanomaterials and monomers or multiple monomers are mixed in a suitable solvent, followed by polymerization with an appropriate reagent to yield polymer nanocomposites (Figure 2). This method enables the fabrication of well-defined multidimensional structures with distinct properties from the initial precursors. Homogenous dispersion in a polymer matrix can be achieved by this technique, which also assists in controlling the size, shape, and morphology of the nanomaterials [2].

#### 2.2.2. Melt Processing

**Figure 3:**
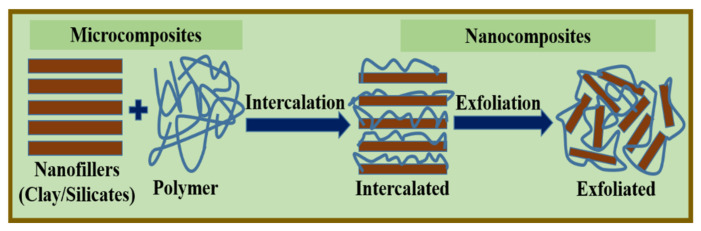
Schematic illustration of a nanofiller/polymer nanocomposite.

The melt processing technique is frequently used for thermoplastic polymers and is recognized as an economically viable, environmentally friendly, and green (solvent-free) technique. In this method, nanofillers or clay materials are incorporated into the polymer matrix by high-temperature annealing and rigorous mixing for a certain duration to encourage the intercalation and exfoliation of silicates, clay, or nanofillers, until a uniform distribution is achieved (Figure 3). The uniform distribution ensures a surface-modification-related compatibility with the host polymer and the processing conditions of the nanofiller [48]. Melt intercalation is a particularly attractive technique because of its durability and compatibility with current polymer processing techniques, such as extrusion and injection molding. This method is environmentally friendly because of the absence of solvents. Moreover, this method permits the use of polymers that cannot be prepared by the in situ polymerization and solution interaction methods [49].

#### 2.2.3. Solution Casting

**Figure 4:**
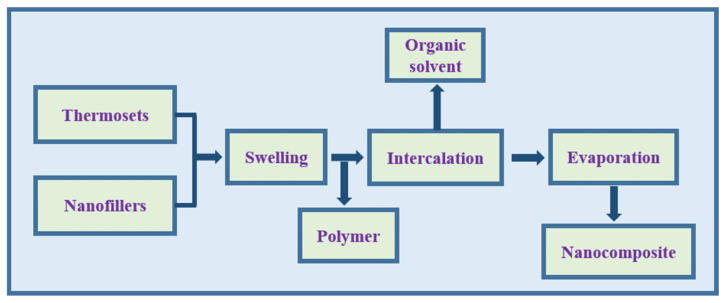
Schematic illustration of solution casting method.

This is the classical method for the production of polymer thin films with distinct thicknesses. In this technique, the polymer is dissolved in a specific solvent by continuous stirring, and the nanofillers are dispersed into the polymer solution to form a homogeneous mixture. This mixture is subsequently cast in a mold to evaporate the solvent and eventually yield thin films with polymer-oriented layers of intercalated clay [2]. Both organic solvents and water can be used to develop nanocomposites with either thermosets or thermoplastics (Figure 4). This method has been widely used to prepare nanocomposites containing water-soluble polymers, such as polyethylene oxide (PEO) and PVOH, and non-aqueous solvent-soluble polymers, including PCL and PLA in chloroform, and HDPE with xylene and benzonitrile solvent [49].

### 2.3. Properties of Polymer Nanocomposites

The properties of polymer nanocomposites based on their microstructure, such as the degree of crystallinity, polar or non-polar groups, the presence of an amorphous phase, and the degree of crosslinking, are correlated with their high aspect ratios, the nature of nanofillers, the chemistry of polymer matrices, and the preparation method. The uniform dispersion of nanofillers in polymer matrices is essential for achieving the desired physical and mechanical characteristics. The barrier, mechanical, thermal, optical, and functional properties of nanocomposites are important parameters in food packaging systems.

#### 2.3.1. Mechanical Properties

The primary intention of packaging is to preserve food from extrinsic deficiencies and influences, such as cracks and random breaks, in the packaging materials. The mechanical properties of polymers, such as strain at break, tenacity, and maximum stress, can be upgraded for use in food packaging systems [50]. Therefore, certain nanofillers are dispersed or reinforced in polymer matrices to improve properties such as strength and stiffness via a reinforcement mechanism [51]. This reinforcement primarily depends on the size, shape, concentration, orientation, surface area, dispersion state, and polydispersity of the nanofillers, possibly resulting in their grafting to the matrix polymers [52]. Nanocomposites prepared with small amounts of fillers exhibit a superior mechanical performance; in this regard, increasing the amount of filler diminishes all of the mechanical properties. These nanofillers can significantly improve the mechanical properties and firmness of the nanocomposites, which in turn alters their relaxation behavior and molecular mobility [53]. Appropriately distributed and aligned clay platelets are remarkably effective in improving the stiffness and mechanical properties of polymer materials, including Young’s modulus (E), strain at break, and stress at break (σ_max_) for polystyrene nanocomposites [54]. An adequate cohesion between the polymer and filler components increases the values of E, σ_max_, and heat resistance, and improves the shear resistance, exfoliation, and corrosion resistance [55]. Different biodegradable polymers reinforced with chitin nanofiber showed higher tensile strength and lower elongation at break values [56].

Particle/polymer matrix interface plays the key role in determining the performance of advanced composite materials such as mechanical properties and dimensional stability. Interfacial adhesion occurs when two different materials such as particle and polymer matrix are blended or combined and create a better dispersion of materials into the matrices. However, the combination of materials must have the same properties, such as hydrophobic fillers and hydrophobic matrices or hydrophilic and hydrophilic materials to achieve a better interfacial adhesion and a strong bond between both materials [57]. Recent studies revealed that the effective mechanical reinforcement of polymeric nanocomposites containing spherical particle fillers is predicted based on a generalized analytical three-phase-series-parallel model [58]. The mechanical properties of particle–polymer matrix composites also depend strongly on the particle size and particle loading. A good adhesion between the fillers and the matrix is a prerequisite for high strength in the resulting composite.

#### 2.3.2. Barrier Properties

Although polymeric materials offer several advantages in the packaging sector, a significant benefit is their intrinsic permeability to small molecules and other gases. Therefore, the loss of quality of packaged food products is due to either oxygen exposure or consistent changes in the movement of water vapor via the walls of the polymer packaging [35]. The permeability of gases and small molecules through the polymer matrices is controlled by various factors, such as the diffusivity, solubility, and morphology of the polymers. Therefore, the barrier properties of polymers are significantly correlated to their intrinsic ability to permit the exchange of low-molecular-weight substances. The structures of nanocomposites and the type and size of nanofillers can affect the degree of modification of the barrier properties of nanocomposites [59]. Well-dispersed nanofillers in the polymer matrix can influence the diffusivity and solubility of the penetrating molecules, particularly in interfacial domains, by increasing the diffusion length and the tortuous path of penetrating molecules to form an impermeable structure in the polymer matrix because of their high aspect ratio [60]. The barrier properties are also affected by the shape, polarity, and crystallinity of the diffusing molecule, the degree of crosslinking, and polymer chains [61]. Improved gas barrier properties and superior permeability have been exhibited by latex membranes and platelet-shaped fillers, respectively, compared to those of the neat membranes [62].

#### 2.3.3. Thermal Properties

Thermal properties are crucial for the use of polymeric materials in a variety of applications, including packaging for consumer products. The low thermal conductivity of polymers or the mismatch between the thermal expansion characteristics of fillers and polymeric components is a major technological barrier [63]. Thermally stable neat polymers typically exhibit thermal conductivities in the range of 0.1–1.4 W/m·K; however, most nanofillers or nanomaterials exhibit high thermal conductivities in the range of 100–400 W/m·K [64]. The use of different types and concentrations of nanofillers plays a vital role in the thermal stability of polymeric nanocomposites, with the nanofillers exhibiting higher *E* (Young’s modulus) values and lower thermal expansion coefficients than those of the polymer components [65]. Nanofillers act as barricades to heat and mass transfer, and reduce the diffusion of gaseous products and the molecular mobility of polymers, which prohibits heat-induced polymer degradation. The combined chemical and physical mechanisms also enhance the thermal stability of polymeric nanocomposites. These routes are the major mechanisms behind the thermal stability of polymeric nanocomposites [66].

#### 2.3.4. Flame Retardancy

The propensity of materials to spread flame away from a fire source must be clearly understood, particularly for several thermoplastic materials, which tend to melt and produce flammable flow or drips, causing fire hazards. Therefore, the flame retardancy of polymeric materials must be improved by the incorporation of flame retardants [67]. Nanofillers such as CNTs and clay are attractive materials as flame retardants because they can concurrently improve the flammability and physical properties of polymeric nanocomposites. However, nanofillers do not exhibit noteworthy fire retardancy on their own, and are therefore combined with other fire retardants [68]. Nanofillers such as nanoclay particles or CNTs can decrease the flammability by prohibiting the vigorous bubbling effect during combustion-induced degradation. The addition of these nanofillers generally leads to the added benefit of improving the physical properties of nanocomposites compared to those of the polymer matrix [69]. Therefore, nanofiller-incorporated nanocomposites can form a constant protective solid layer consisting of clay particles and carbonaceous char (CNTs) on the burning surface [70].

#### 2.3.5. Optical Properties

The incorporation of nanomaterials into polymer matrices provides the possibility of remarkable improvements in the optical properties of polymeric nanocomposites. The unique optical properties of nanomaterials are associated with the effects of dielectric restriction, dimensional quantization, and the excitation of local surface plasmons [71]. The spectral position and intensity of surface plasmon resonance (SPR) are extremely specific for various nanomaterials and strongly depend on both the spatial organization and properties of nanomaterials or nanofillers. The optical properties of nanocomposites can be regulated by altering the size, shape, and concentration of nanomaterials, as well as the dielectric constant of the polymer matrix [72]. The linear and non-linear optical properties of nanofiller-infused nanocomposites are influenced by the excitation of local SPR, especially the collective oscillations of the conduction electrons. The plasmon properties of nanocomposites are categorically related to their sub-micrometer-scale ordering [73]. Moreover, the addition of graphene and CNTs provides certain benefits to other useful optical properties.

## 3. Biodegradable Polymers (Biopolymers)

Petroleum-based polymers have the ability to satisfy all the packaging requirements of the food industry. However, they are non-biodegradable, non-renewable, or non-compostable, which can lead to serious issues related to disposal and waste generation worldwide, consequently leading to environmental damage. Therefore, the research on packaging must be focused on promoting and developing bio-based plastics that are alternatives or replacements for fossil fuels or synthetic polymers to effectively minimize waste disposal [74]. Biodegradable materials are capable of undergoing biological decomposition or degradation to yield water, methane, carbon dioxide, inorganic compounds, and biomass by enzymatic activities of microorganisms, depending on the environmental conditions of the process. Biopolymers are biodegradable polymers that comprise covalently bonded monomeric units, which construct chain-like molecules that can be degraded or metabolized by naturally occurring microbes [75]. Biodegradable polymers are typically derived from animal sources, agricultural feedstock, marine and food processing industrial wastes, or microbial sources, including starch, proteins, peptides, DNA, and RNA.

**Figure 5:**
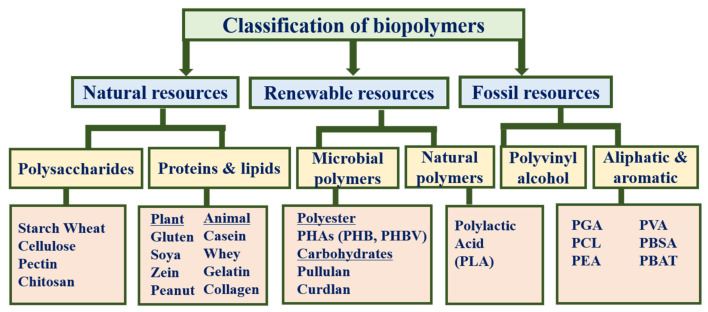
Classification of biopolymers for food packaging applications.

Biopolymers can be classified into the following groups based on the origin of the raw materials (renewable or non-renewable) and their manufacturing process (Figure 5): natural resources, microbial or renewable resources, and synthetic or fossil resources. Natural biopolymers are subdivided into polysaccharides (starch, wheat, cellulose, pectin, and chitosan) and proteins and lipids (gluten, soya, zein, peanut, casein, whey, gelatin, and collagen). Similarly, renewable polymers are categorized into microbial polymers, such as polyesters (PHB and PHBV), carbohydrates (pullulan and curdlan), and natural polymers, such as PLA. Synthetic polymers are further categorized into PVA and aliphatic and aromatic polymers (polyglycolic acid (PGA), PCL, polyester amides (PEAs), PVA, poly (L-lactide) (PLA), polybutylene adipate-*co*-terephthalate (PBAT), and polybutylene succinate-*co*-butylene adipate (PBSA)) [76,77].

Biopolymers are regarded as the most promising materials for food packaging applications. However, they generally exhibit poor barrier and mechanical properties with regard to processing ability and end-use applications. In particular, the high gas and vapor permeability, brittleness, low heat-distortion temperature, and poor resistance to protracted processing operations of biopolymers significantly limit their industrial applications [78]. However, both natural and synthetic nanofillers can be used to improve their physical and mechanical properties. Fully biodegradable nanocomposites can be produced using polymer matrices and fillers derived from renewable resources [79]. Various examples of biopolymers used as packaging materials are listed in Table 1.

The plastic industry promised to be a boon compared to other industries in its initial stages. Recent research and developments focused on the production and optimization of bio-derived products from various plant matters or biomass in a sustainable and economical way. Nevertheless, biopolymers occupy their own position and have the largest market share in plastic industries. Besides biodegradable plastics, natural biopolymers from polysaccharides and polypeptides have also been widely used for their biostability, sustainability, mechanical properties, biocompatibility, and minimum cytotoxicity in food packaging and other multifarious applications [99]. However, the future prospects of biopolymers seem to be the most promising way to enhance their mechanical and thermal properties. Biopolymers are not likely to replace all fossil fuels for packaging applications, where the cost of biopolymers need to be looked into objectively and addressed in light of environmental issues [100]. Most biopolymers are costly to produce, and since petroleum based polymers are cheaper, industries use them without considering the environmental factors. Furthermore, economic concerns must be addressed, as the future of all biopolymer products depends on their cost competitiveness, by-products, and social impact [101].

## 4. Applications of Bio-Based Nanocomposites for Food Packaging Systems

The incorporation of functional nanomaterials into polymer matrices can assist in the development of food packaging materials with improved mechanical and barrier properties. Moreover, the fundamental properties of packaging materials, such as flexibility, durability, resistance to temperature and humidity, and flame resistance, can be further altered by the addition and modification of different nanomaterials to improve the shelf life and quality of the food products [102]. Different natural and inorganic–organic nanofillers, including cellulose nanocrystals, zein NPs, and cellulose NPs, and inorganic nanomaterials such as clay and layered silicates (montmorillonite), mesoporous silica nanoparticles (MSNs), metal and metal oxide NPs (Ag, Au, Cu, ZnO_2_, SiO_2_, TiO_2_, Fe_2_O_3_, and Al_2_O_3_), layered double hydroxides, nanotubes (CNTs), fullerenes, nanorods, and salts, are typically employed as nanoreinforcements [103]. Among the various types of food packaging materials, edible coatings or edible films in the form of films or thin layers are used to shield the food products and create a mass-transfer barrier. Edible coatings are more relevant for direct application to food products, whereas non-edible coatings are used as protective containers. The application of edible coatings can be found in the agriculture, bakery and cheese, and meat processing industries to furnish color, enzymes, flavors, antioxidants, and anti-browning compounds to food products [102].

Clay and silicates are natural inorganic compounds with variable chemical compositions, relative simplicity, and low cost, which have attracted research attention as potential nanomaterials owing to their availability and barrier, mechanical, and thermal properties. The combination of clays/silicates and polymers yields superior barrier properties and lengthens the diffusive path for infiltrating molecules. Nanoclays can be categorized into several subclasses, including montmorillonite (MMT), kaolinite, bentonite, halloysite, hectorite, sepiolite, and cloisite [103]. Prior to their incorporation into polymers, natural clays have been modified with organic compounds such as tetra-alkyl ammonium salts and alkyl amine to generate an intercalated and exfoliated mixed structure, which afforded superior properties to those of the original polymers [104]. Intercalated nanocomposites exhibit a multilayered structure with alternating nanofiller/polymer layers separated by a few nanometers; moreover, the exfoliated nanocomposites exhibit comprehensive polymer penetration with random dispersion of clay layers [105]. MMT clay has been extensively investigated for developing nanocomposites with a variety of polymers, such as nylon, PE, PVC, and starch. The amounts of incorporated nanoclays typically vary from 1% to 5% by weight and are one dimension smaller than 1 nm. The use of nanocellulose is considered an advanced approach for the preparation of sustainable food packaging in the form of both coatings and fillers. Additionally, nanocellulose fibers have been designed or modified to enhance their interaction with the matrix phase and improve the intrinsic properties of active and intelligent packaging systems [106].

CNTs, such as single-wall carbon nanotubes (SWCNTs) or multi-walled carbon nanotubes (MWCNTs), have also been incorporated into various polymers such as PVA, PA, and PET. CNTs also exhibit antibacterial properties, which are associated with their direct penetration of microbial cells and chemiresistive sensing [107]. Starch has been extensively studied as a substitute material for food packaging applications because of its biodegradability, availability, and non-toxicity, in addition to its stability in air. Moreover, starch enhances the tensile strength and modulus of pullulan films, with the positively charged ions present on the surfaces of these antimicrobial agents contributing to their antimicrobial action [108]. Chitosan is a natural cationic polymer known for its biocompatibility, biodegradability, non-toxicity, low cost, hydrophilicity, and antimicrobial activity, and is considered a potential polymer for food packaging, especially in the form of edible films and coatings [109]. Chitosan/polyethylene active antibacterial bags showed potential in inhibiting the activities of total mesophilic bacteria, molds, coliforms, and yeasts in chicken drumsticks, and in maintaining the color, pH, and hardness of samples [110]. Similarly, various metal and metal oxide NPs, such as Au, Ag, Cu, Zn, ZnO, TiO_2_, SiO_2_, and MgO, have been examined for diverse active-food-packaging applications. The properties of metal and metal oxide nanomaterials, such as mechanical strength, thermal and chemical stability, gas and water barrier properties, heat resistance, biodegradability, and active antimicrobial activities, have led to an improved performance in active food packaging applications [111]. Various types of nanocomposites used in food packaging applications are listed in Table 2.

### Active and Intelligent Packaging Systems

Active and intelligent packaging are two forms of smart packaging that have been recently developed to enable the marketing of food products and provide passive protection against environmental conditions and contamination to extend the shelf life of food products. An active packaging material is a neat or modified substance that increases the shelf life of food products or enhances their safety or sensorial properties to maintain their quality [129]. Active packaging materials interact with packaged food and the environment in a certain manner and/or react to various stimuli, owing to their intrinsic properties or the incorporation of certain special additives in the packaging material to maintain the food quality [130]. Various categories of active food packaging exist, such as antimicrobial, antioxidant, oxygen scavenging, ethylene scavenging, liquid, moisture, odor, flavor absorbing, and ultraviolet barrier [131]. However, unlike intelligent (responsive) packaging, active packaging does not react to a specific trigger mechanism.

Intelligent packaging materials apply intelligent functions related to responsive packaging throughout the food supply chain, which include locating, registering, detecting, communicating, monitoring, and applying scientific logic. Therefore, these materials can trigger alerts for consumers by detecting microorganisms and food spoilage, extend shelf life, ease decision-making, improve the quality and safety of food, provide information, and warn of possible problems [132]. In addition, intelligent packaging can enable the release of antimicrobials, antioxidants, and other compounds upon detecting food spoilage under specific environmental changes to extend the shelf life of food products [133]. As discussed previously, the reinforcement of polymer matrices by nanofillers can improve the mechanical, barrier, and thermal properties of food packaging materials. Additionally, agents such as antimicrobials, antioxidants, nutraceuticals, coloring agents, flavors, and biosensors have been added to polymer matrices to enhance their smart functions, with regard to the quality, stability, and safety of food products [134]. Recently, Taherimehr et al. discussed the trends and challenges of biopolymer-based nanocomposites for food packaging applications [135]. However, the current review focused more on natural and inorganic–organic nanomaterials, clay, layered silicates, mesoporous silica nanoparticles, metal and metal oxide nanoparticles, layered double hydroxides, nanotubes, fullerenes, and nanorods employed as nanoreinforcements to develop nanocomposites for active and intelligent food packaging applications.

## 5. Antimicrobial Properties of Bio-Nanocomposites

Antimicrobial packaging is a robust technology that protects packaged food products from spoilage, which can occur via contamination by food-borne pathogens (bacteria, parasites, and viruses), leading to food-borne diseases [136]. This type of packaging can be prepared either by applying a coating layer (antimicrobial agent) within the packaging material or by incorporating an antimicrobial agent into the packaging material. Antimicrobial packaging of food products is a type of active packaging owing to the use of antimicrobial agents, growth inhibitors, and antimicrobial carriers [23]. Nanomaterials or nanocomposites, owing to their enhanced surface reactivity, high surface-to-volume ratio, and physicochemical and antimicrobial properties, impede the activity of microorganisms more efficiently than their micro- or macro-scale counterparts. The efficacy and performance of active food packaging have been enhanced through nanoencapsulation or the incorporation of natural antimicrobial-loaded nanocarriers [137]. The most commonly used antimicrobial nanocomposite materials include metal and metal oxide NPs (such as Au, Ag, Cu, ZnO, MgO, and TiO_2_), natural biopolymers (chitosan), organic nanoclay (Ag-zeolite and MMT), enzymes (peroxidase and lysozyme), natural bioactive compounds (thymol, carvacrol, nisin, and isothiocyanate), and synthetic agents (EDTA, ammonium salts; benzoic, propionic, and sorbic acids) [23].

Different species of Gram-negative and Gram-positive bacteria, including *Salmonella* spp., *Staphylococcus aureus*, *Staphylococcus epidermis*, *Escherichia coli*, *Listeria monocytogenes*, *Pseudomonas aeruginosa*, *Enterococcus faecalis*, *Vibrio cholera*, and *Bacillus cereus*, are responsible for food spoilage. In addition, *Aspergillus* and *Rhizopus* (molds), and *Candida* and *Torulopsis* (yeasts) are also involved in foodborne infections [138]. A few recently reported antibacterial activities of metal and metal oxide nanocomposites are briefly described in the following. A carboxymethyl cellulose film coated with AgNPs exhibited antibacterial efficacy against *S. aureus* and *E. coli* [139]; cellulose acetate with AgNPs [140]. AuNPs with bacteriocin inhibited the activities of *E. coli*, *B. cereus*, *S. aureus*, and *Micrococcus luteus* [141]. Gellan gum-sodium carboxymethyl cellulose ((GC)-SiO_2_) and GC-SiO_2_-octadecyldimethyl-(3-trimethoxysilylpropyl)-ammonium chloride (ODDMAC) nanocomposites were effective against *B. cereus* and *S. aureus* [142]. Chitosan-ZnO coatings reduced the initial numbers of *E. coli* in white-brined cheese [143]. Table 3 provides a detailed list of select investigations that have examined the antimicrobial properties of different nanofillers.

### Mechanisms of Antimicrobial Action

**Figure 6:**
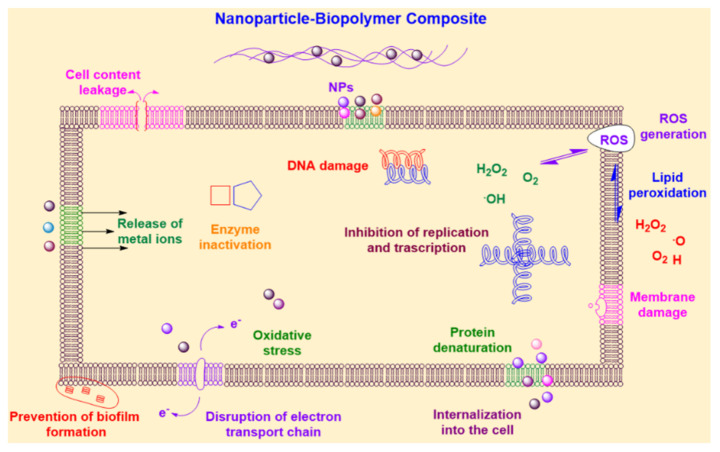
Schematic representation of antimicrobial mechanisms of action of nanocomposites designed for food packaging.

Nanomaterials or nanocomposites can be employed as antimicrobial agents, growth inhibitors, antimicrobial carriers, and antimicrobial packaging films. Antimicrobial bio-nanocomposite films are applied as food packaging materials primarily for cheese, meat, bread, fish, poultry, vegetables, and fruits [170]. These nanocomposite materials have potent antibacterial effects through various mechanisms of action that interact precisely with microbial cells, including the disruption of cell walls, interruption of transmembrane electron transfer, oxidation of cell components, formation of reactive oxygen species (ROS), disruption of enzyme activity, destruction of internal cell organelles, prevention of DNA synthesis, and cellular death [171]. The probable mechanisms of antimicrobial action of the nanocomposites developed as active food packaging materials are illustrated in Figure 6.

Nanocomposites have a remarkably positive zeta potential, which promotes their interaction with cell membranes by electrostatic binding to cell walls and releasing metal ions. The negatively charged bacterial membranes and positively charged nanocomposites induce electrostatic attraction and modify the permeability of the cell membrane. Therefore, disrupting the integrity of bacterial membranes is an efficient mechanism of action [172]. ROS production is an alternative mechanism that affects the physiological functions of cells and eventually damages DNA. Different types of ROS, such as superoxide anionic radicals, hydroxyl radicals, hydrogen peroxide, and the hydroxyl radical produced in mitochondria, exhibit varying levels of activity. Protein dysfunction is another mechanism of action by which nanocomposites bind to cytosolic proteins, such as DNA and enzymes, which leads to oxidative stress, damage of communication channels, peroxidation of cellular constituents, DNA strand breakage, lipid peroxidation, and modification of nucleic acids [15]. However, in the case of enzymes, carboxylation results in the loss of catalytic activity and accelerates protein degradation [173].

## 6. Conclusions and Future Trends

Food packaging plays a critical role in protecting food products from external contamination and maintaining their quality, integrity, and safety throughout their shelf life. Synthetic-polymer-based materials are predominantly used as packaging materials in the food industry because of their ease of production, versatility, affordability, functionality, and properties of low weight, flexibility, and low cost. However, these synthetic polymers are non-degradable, and most of the plastic waste and debris heavily pollute the environment. This necessitates the development and use of biodegradable polymer materials to resolve these environmental problems. Bio-based polymers or renewable-resource-based biopolymers, such as cellulosic plastics, starch, corn-derived plastics such as PHAs, and PLA are sustainable, high-performance materials with tremendous potential for replacing conventional petroleum-based food packaging materials. However, biopolymers have certain disadvantages compared to synthetic polymers, such as inferior thermal and mechanical properties (tensile strength and brittleness), moisture sensitivity, and water-vapor barrier performance.

The use of nanotechnology in the food sector can ensure food quality and safety by enhancing the potency of food packaging and the shelf life of food products. The application of nanotechnology to develop novel food packaging functions can enable enhancements in the properties of food, such as taste, healthiness, and nutritiousness via the packaging. The incorporation of nanoparticles or nanofillers in food packaging materials can improve various properties of biopolymers, such as mechanical properties, barrier properties against water and oxygen, protection against UV radiation, absorption of moisture, release of antimicrobials, and other environmental factors. The biodegradability of these nanocomposites can be modified by selecting appropriate polymers and nanomaterials to yield desired properties and enable their application in food packaging. In addition, biologically active substances, such as antimicrobials, growth inhibitors, and antimicrobial carriers, can be added to enable the desired functional properties. These nanocomposites can be modified by incorporating either organic or inorganic antimicrobial agents that exhibit excellent antibacterial activity against both Gram-positive and Gram-negative food-borne pathogens. The inclusion of antibacterial NPs, such as Ag, MgO, ZnO, TiO_2_, graphene, and carbon dots, in bio-nanocomposite films enables their use as new active packaging materials to improve the quality and safety of food products over a longer period. Nanosensors for intelligent packaging can be designed to control the internal (detecting microorganisms and chemicals in the packaging) and external conditions of food products (detecting atmospheric influences).

The present review provides information related to the development of bio-based polymeric nanocomposites to improve the quality, safety, and shelf life of packaged food products. However, further research should focus on the effects of combinations of nanomaterials, such as bi-, tri-, and multi-metallic nanocomposites, to achieve better results. In addition, the molecular interactions of biopolymers with food matrices and the formulations of nanomaterials in polymer matrices to minimize organoleptic effects should be investigated. Furthermore, standard methods and probable toxicity evaluations of nanofillers and biopolymers should be established. Current toxicity tests have revealed that the toxicity of nanomaterials primarily depends on their size, shape, surface-to-volume ratio, doping concentration, and duration. Future research on active packaging must focus on improving the safety of nanomaterials, owing to the limited studies on the possible toxic influence of these packaging films. In addition, health and safety aspects, the management of environmental issues, and a hazard and risk assessment must be considered prior to their application as safe and effective food packaging materials. The use of these modern and alternative preservation techniques can significantly inhibit pathogens, extend the shelf life, and fulfill consumer demands such as the high quality, convenience, safety, freshness, taste, aroma, color, and texture of packaged food. These biopolymer-based nanocomposites exhibit tremendous potential for a wide range of applications in the food industry as sustainable, cost-effective, active, and intelligent packaging materials for food preservation.

## Figures and Tables

**Table 1:** Biopolymers with different properties used as packaging materials in the food industry.

Biopolymers	Source	Properties	Applications	Ref.
Cellulose	Agricultural waste	Highly crystalline, chemically and thermally stable, antimicrobial properties	Biodegradable packaging, microencapsulation	[80,81,82]
Starch	Potato, corn, wheat	Enhanced gas barrier and consistent with antioxidant and antimicrobial properties	Encapsulation and biodegradable packaging	[83,84]
Pectin	Apple pomace and citrus peels	Biodegradability, biocompatibility, edibility, and versatile physical and chemical properties	Biodegradable films for food packaging and microencapsulation	[85]
β-D-glucan	Oat and barley	Rheological, biocompatibility and biodegradable properties	Encapsulation matrix and for film-forming preparations	[86]
Chitosan	Crab, shrimp, crawfish	Moderate mechanical strength, low barrier properties, inherent antimicrobial properties	Biodegradable films, and microencapsulation	[83,87,88]
Gums	Acacia tree	Excellent adhesive strengths, enhanced structural, thermal and gas barrier properties	Adhesive packaging applications	[89]
Alginate	Marine brown algae	Low oxygen permeability, vapors, flexibility, and water solubility	Intelligent and green packaging technologies	[89]
Agar	Marine red algae	High transparency, permeability, thermal stability, or mechanical strength of the film	Food packaging applications	[90]
Carrageenan	Cell walls of seaweeds	Enhancing sensory properties, reducing moisture loss	Edible biodegradable films and coatings	[91]
Casein	Milk, yogurt and cheese	Biodegradability, high thermal stability, non-toxicity	Protein-based coatings and films in food packaging	[92]
Whey	Milk, yogurt and cheese	Excellent barrier characteristics for oxygen, oil, and aroma	Biodegradable films for food packaging	[93]
Gelatin	Cattle bones	Enhanced mechanical, and optical, barrier effect against gas flow	Gelatin-based coatings and films for food packaging	[94]
Zein	Corn protein	Good barrier properties, high compatibility	Bio-based packaging and edible coatings	[95]
Soy proteins	Soybeans	Remarkable gas barrier and weaker mechanical properties, better antimicrobial properties	Biodegradable films and microencapsulation	[96]
Collagen	Fish skin, bones, fins	Improved rheological properties, high-water absorption capacity	Smart and active packaging.	[97]
Wheat gluten	Wheat flour	Improved structural, surface, gas barrier, and water vapor properties	Paper coating and food packaging	[98]

**Table 2:** Examples of nanofillers and polymer matrices that have been applied as nanocomposites in food packaging systems.

Nanofillers	Polymer Matrix	Properties	Applications	Ref.
Cellulose nanocrystals	PLA	Oxygen barrier	Used as polar and non-polar simulants in food packaging materials	[112]
Cellulose nanocrystals	PLA	Mechanical and antimicrobial	Biocidal activity in food packaging industry	[113]
Organoclay	LDPE and HDPE	Rheological and barrier	Oxygen permeability of polymer decreasing slowly with increases in clay concentration	[114]
Starch nanocrystals	Potato starch	Mechanical and thermal	Biodegradable edible films for packaging	[115]
MMT	PCL	Mechanical	Biodegradable polymer nanocomposites for food packaging	[116]
Clay ZnO	PEA starch	Mechanical strength	Medical, agriculture, drug release, and packaging fields	[117]
Zein NPs	WPI (whey protein isolate)	Mechanical, water vapor barrier	Effective food packaging materials.	[118]
MMT	WPI	mechanical	WPI film for food packaging	[119]
Anionic sodium MMT	PET	Oxygen transmission rate decreased	Replacement of aluminum foil in food packaging systems	[120]
Cellulose whiskers	PEA starch	Tensile, thermomechanical	Biodegradable edible films for packaging	[121]
Cellulose nanocrystals	PLA	Mechanical and oxygen barrier	Biomaterial for food packaging systems.	[122]
MMT	Cellulose acetate	Mechanical	Replacing oil-based high performance plastics for food packaging	[123]
Starch nanocrystals	Polyurethane	Mechanical	Biomaterial for food packaging systems	[124]
Bacterial cellulose nanoribbons	Chitosan	Mechanical	New materials for the food packaging	[125]
Chitosan–tripolyphosphate NPs	Hydroxypropyl methylcellulose	Mechanical and barrier properties	Improved functionality to edible films for food packaging	[126]
Chitin whiskers	Starch	Mechanical, water vapor barrier	Improved properties to prolong the shelf life of packaged foods	[127]
Graphene	Poly(methyl methacrylate)	Heat resistant and barrier properties	Promising material for food packaging systems	[128]

**Table 3:** Examples of bio-based nanocomposites investigated for their antimicrobial properties.

Nanomaterials	Biopolymer	Pathogens	Applications	Ref.
Ag	Chitosan	*E. coli*, *Solmonella*, *S. aureus*	Active and intelligent food packaging	[144]
Ag	LDPE	*E. coli*, *E. faecalis*, *S. aureus*	Improved food quality and safety	[145]
Ag	Cellulose	*E. coli*, *S. aureus*	Potential bacterial barrier in food packaging	[146]
Au	PVA	*E. coli*	Active food packaging for banana fruits	[147]
CuS	Agar	*E. coli*, *L. monocytogenes*	Active food packaging	[148]
CuO	Agar, alginate, chitosan	*E. coli*, *L. monocytogenes*	UV-screening and food packaging	[149]
ZnO	Gelatin, cellulose	*E. coli*, *L. monocytogenes*, *S. aureus*	Active food packaging	[150]
TiO_2_	Chitosan	*E. coli*, *S. aureus*	Active multifunctional food packaging	[151]
ZnO	Carboxymethyl cellulose	*E. coli*, *S. aureus*	Active food packaging	[152]
SiO_2_	PHBV	*E. coli*, *S. aureus*	Eco-friendly, cost-effective food packaging materials.	[153]
SO_2_	PA, PE	*E. coli, L. monocytogenes*, *S. aureus*	Active packaging for selected types of foods	[154]
ZnO	Soy protein isolate	*Aspergillus niger*	Ideal packaging matrix for food preservation	[155]
TiO_2_	Zein, sodium alginate	*E. coli*, *S. aureus*	Improved shelf life and quality of food stuffs	[156]
MgO	PLA	*E. coli*	UV-screening and active food packaging	[157]
Carbon dots	Bacterial nanocellulose	*E. coli*, *L. monocytogenes*	UV-screening and forgery-proof packaging	[158]
SiO_2_	Chitosan	*E. coli*, *S. aureus*, *S. typhimurium*	Active food packaging	[159]
CNTs	Allyl isothiocyanate	*Salmonella* spp.	Active packaging for shredded cooked chicken	[160]
MWCNTs	Chitosan, PLA	*E. coli*, *S. aureus*, *B. cinerea*, *Rhizopus*	Active packaging for fruits and vegetables	[161]
MSN	PHBV	*E. coli*, *S. aureus*	Interlayers or coatings for active food packaging	[162]
Cellulose	Agar	*E. coli*, *L. monocytogenes*, *S. aureus*	Active packaging for safety and shelf-life of food	[163]
Halloysite	Starch	*C. perfringenes*, *S. aureus*, *L. monocytogenes*	Active and useful barrier to control food contamination.	[164]
Chitosan	Fish gelatin	*S. aureus*, *L. monocytogenes*, *S. enteritidis*, *E. coli*	Greater flexible films, with decrease in water vapor permeability	[165]
MMT	Chitosan	*L. monocytogenes*, *E. coli*, *P. putida*	Antioxidant and antibacterial films for food preservation	[166]
Cinnamaldehyde nanoemulsions	Pectin, papaya puri	*E. coli*, *L. monocytogenes*, *S. aureus*	Environmentally friendly antimicrobial packaging material for food applications	[167]
Cellulose nanofiber	Starch	*B. subtilis*, *E. coli*	Biopolymer active food packaging	[168]
PLA nanofibers	PLA	*E. coli*, *S. aureus*	Effectively prolong the shelf-life of pork.	[169]

## Data Availability

Not applicable.
